# Bilirubin influences the predictive effect of body mass index on hospital mortality in critically ill patients

**DOI:** 10.1016/j.heliyon.2024.e32089

**Published:** 2024-05-29

**Authors:** Xiao-Ling Lv, Ying-Xing Yue, Bing-Bing Jia, Ying-Zheng Weng, Yan Lu, Zhou-Xin Yang

**Affiliations:** Zhejiang Key Laboratory of Geriatrics and Geriatrics Institute of Zhejiang Province, Zhejiang Hospital, 1229 Gudun Road, Hangzhou, 310030, China

**Keywords:** Body mass index, Total bilirubin, Hospital mortality, Critically ill patients

## Abstract

**Introduction:**

Body mass index (BMI) can predict mortality in critically ill patients. Moreover, mortality is related to increased bilirubin levels. Thus, herein, we aimed to investigate the effect of bilirubin levels on the usefulness of BMI in predicting mortality in critically ill patients.

**Methods:**

Data were extracted from the Medical Information Mart for Intensive Care (MIMIC IV) database. Patients were divided into two groups according to their total bilirubin levels within 24 h. Cox proportional hazard regression models were applied to obtain adjusted hazard ratios and 95 % confidence intervals for the correlation between BMI categories and hospital mortality. The dose–response relationship was flexibly modeled using a restricted cubic spline (RCS) with three knots.

**Results:**

Of the 14376 patients included, 3.4 % were underweight, 29.3 % were of normal body weight, 32.2 % were overweight, and 35.1 % were obese. For patients with total bilirubin levels <2 mg/dL, hospital mortality was significantly lower in patients with obesity than in normal body weight patients (p < 0.05). However, the opposite results were observed for patients with total bilirubin levels ≥2 mg/dL. The Cox proportional hazard regression models suggested that the risk of death was lower in patients with overweightness and obesity than in normal body weight patients when the total bilirubin levels were <2 mg/dL, but not in the other case (total bilirubin levels ≥2 mg/dL). RCS analyses showed that, for patients with total bilirubin levels <2 mg/dL, the risk of death gradually decreased with increasing BMI. Conversely, for patients with total bilirubin levels ≥2 mg/dL, this risk did not decrease with increasing BMI until reaching obesity, after which it increased rapidly.

**Conclusion:**

BMI predicted the risk of death differently in critically ill patients with different bilirubin levels.

## Abbreviations

BMI: Body Mass Index
CIs: Confidence Intervals
HRs: Hazard Ratios
ICD: International Classification of Disease
ICU: Intensive Care Unit
LOS: Length Of Stay
MIMIC Ⅳ: Medical Information Mart for Intensive Care Ⅳ
RCS: Restricted Cubic Spline

## 1 Introduction

Body mass index (BMI) is an essential indicator used to evaluate whether people are obese, overweight, or underweight. Normal BMI is beneficial to survival. Being underweight may increase the risk of mortality, whereas being overweight and obese often lead to the development of various complications, such as cardiovascular and cerebrovascular diseases [1,2] and increased probability of sepsis [3]. The risk of death can be predicted in critically ill patients by calculating their BMIs. A study on critically ill patients showed that, when compared with patients with normal body weight, those underweight had increased mortality, whereas those overweight and obese had decreased mortality [4].

Nevertheless, the protective effect of overweightness and low degree of obesity on critically ill patients remains unclear. One speculation is that the excess fat stored in the overweight or obese state can be metabolized when necessary to maintain patient survival. The liver plays a crucial role in fat metabolism, and the digestion, absorption, and catabolism of lipids are closely associated with liver functions [5]. Liver failure is an important factor in the death of intensive care unit (ICU)-admitted critically ill patients accompanied by various underlying diseases [6]. Liver damage results in dysfunctional fat metabolism, which, in turn, affects fat transformation into substances such as carbohydrates to provide energy.

Bilirubin is a metabolite of the red blood cell heme, which can be metabolized by the liver [7]. Bilirubin levels increase in case of abnormal liver functioning, and a high bilirubin level is an index of critical liver function [8]. Bilirubin level ≥2 mg/dL is considered abnormal [9,10]. Additionally, increased total bilirubin level is an important risk factor for several ICU-borne diseases, such as sepsis [11] and acute respiratory distress syndrome [12]. We previously investigated the relationship between total bilirubin levels in the serum and clinical outcomes in critically ill patients and found that the mortality rate of patients with total bilirubin levels <2 mg/dL was lower than that of patients with total bilirubin levels ≥2 mg/dL. Even after propensity score matching, the mortality of patients with total bilirubin levels ≥2 mg/dL was high [13]. Moreover, the length of stay (LOS) in the hospital of patients with total bilirubin levels <2 mg/dL was shorter than that of patients with total bilirubin levels ≥2 mg/dL [13]. Therefore, monitoring the total bilirubin level of critically ill patients is crucial for predicting their clinical outcomes.

Herein, we speculated that the ability of BMI to predict mortality in critically ill patients was affected by the total bilirubin levels. Accordingly, we assigned the patients into two groups based on their total bilirubin levels as ≥ 2 mg/dL and <2 mg/dL. Differences in their clinical outcomes at different BMIs were compared to determine the effect of total bilirubin levels on the ability of BMI to predict mortality in these patients.

## 2 Methods

### 2.1 Data extraction

This study included adult patients from the Medical Information Mart for Intensive Care IV (MIMIC IV) database [14], which contained more than 70000 records of patients admitted to the ICU in Beth Israel Deaconess Medical Center (Boston, MA, USA) from 2008 to 2019. Patient-related data were collected by the hospital. Informed consent was waived off as the identities of these patients were concealed. The author ZX Y extracted the data from the database after completing the training course of the Collaborative Institutional Training Initiative.

### 2.2 Study subjects and clinical outcomes

As we investigated the predictive ability of BMI in critically ill patients with different bilirubin levels, adult patients admitted to the ICU were included in this study. Patients with no BMI record or weight <10 kg were excluded. Patients without total bilirubin level records within 24 h after admission were excluded. For patients with multiple ICU admissions, only the first record of ICU admission was considered. Patients with ICU stay of <24 h were excluded. Based on the total bilirubin level within 24 h after admission, the patients were divided into the following two groups: <2 mg/dL and ≥2 mg/dL. Underweight (BMI <18.5 kg/m^2^), normal body weight (18.5 kg/m^2^ ≤ BMI <25 kg/m^2^), overweight (25 kg/m^2^ ≤ BMI <30 kg/m^2^), and obese (BMI ≥30 kg/m^2^) individuals were defined as previously reported [15]. Hospital mortality was determined based on the time of death, as extracted from the MIMIC IV database, and whether the patient died in the hospital. Next, variables with missing values < 5 % were replaced by median values and variables with missing values > 5 % were not imputed. Hospital mortality was the primary outcome indicator, and the hospital and ICU LOSs were the secondary outcome indicators.

### 2.3 Statistical analysis

The normality of data distribution was evaluated by using the Kolmogorov–Smirnov test and by visually examining histograms. Owing to the skewed distribution of continuous variables, data were expressed as the median (25th, 75th percentile). The Kruskal–Wallis test was performed for non-parametric analysis in order to compare the four BMI groups. Furthermore, a post-hoc pairwise comparison-adjusted by the Bonferroni correction was performed between the BMI groups. Categorical variables were presented as numbers and percentages, and differences among the BMI groups were assessed using the Chi-square test, followed by post-hoc tests with the Bonferroni correction. Cox proportional hazard regression models were used to obtain adjusted hazard ratios (HRs) and 95 % confidence intervals (CIs) to explore the association between the BMI groups and hospital mortality. HRs were adjusted for gender, age, alanine aminotransferase (ALT), aspartate aminotransferase (AST), and comorbidities, including diabetes, renal failure, congestive heart failure, chronic pulmonary disease, and liver diseases. Multivariable linear regression models were used to evaluate the independent predictive ability of BMI on the hospital and ICU LOSs by adjusting for potential confounders. Estimates and 95 % CIs were calculated. Multicollinearity was determined using the variance inflation factor (VIF) method, with a VIF ≥5 indicating multicollinearity. In regression analyses, nominal independent variables with more than two categories were defined as dummy variables. The dose–response relationship was flexibly modeled using a restricted cubic spline (RCS) with three knots to investigate the potential nonlinear relationship between BMI (continuous variables) and hospital mortality. Statistical analyses were performed using SPSS 22.0 (IBM Corp., Armonk, NY, USA) and the statistical package R (The R Foundation, http://www.r-project.org, version 4.3.0). p < 0.05 was considered to indicate statistical significance.

## 3 Results

### 3.1 Baseline characteristics

**Fig. 1:**
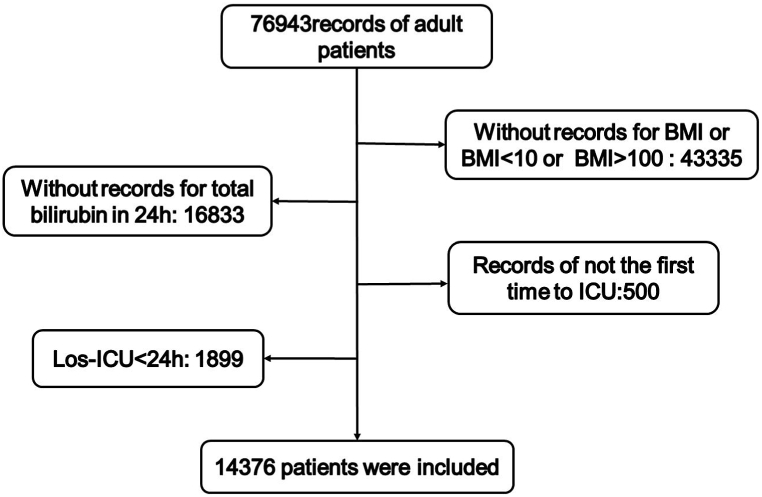
Flowchart depicting the patient selection process.

In total, 76,943 adult patient records were extracted from the MIMIC IV database, and 43,335 records were excluded because of missing BMI data or BMI <10 or BMI >100 (Fig. 1). Furthermore, 16,833 records were excluded because bilirubin records within 24 h were unavailable, 500 records were excluded because they did not enter the ICU for the first time, and 1899 records were excluded because they were admitted to the ICU for less than 24 h. Finally, 14,376 patients were included in the study.

The study patient characteristics are presented in Table 1. Among the patients, 490 (3.4 %) were underweight, 4207 (29.3 %) were of normal body weight, 4631 (32.2 %) were overweight, and 5048 (35.1 %) were obese. Furthermore, 49.6 % of the underweight patients were women, whereas the proportion of men was higher than women in the remaining three groups. In addition, patients with obesity were younger than the patients in the other three groups. The total bilirubin levels were lower in the underweight group than in the remaining three groups.

**Table 1:** Clinical characteristics of critically ill patients by BMI categories.

Variables	Total	Underweight	Normal body weight	Overweight	Obesity	p value
Number of patients	14376(100.0 %)	490(3.4 %)	4207(29.3 %)	4631(32.2 %)	5048(35.1 %)	–
BMI (kg/m^2^)	27.47(23.83,32.32)	17.31(16.19,17.97) *	22.60(20.99,23.88)	27.31(26.13,28.52) *	34.68(31.96,39.15) *	<0.001
Gender (male)	8477(59.0 %)	243(49.6 %) *	2392(56.9 %)	2970(64.1 %) *	2872(56.9 %)	<0.001
Age (year)	65.78(54.29,76.83)	67.20(55.02,78.70)	67.11(54.25,80.17)	67.15(55.50,78.11)	63.84(53.26,73.21) *	<0.001
Maximum bilirubin (mg/dL)	0.70(0.40,1.40)	0.50(0.30,0.90) *	0.70(0.40,1.30)	0.70(0.40,1.50) *	0.70(0.40,1.60) *	<0.001
Maximum ALT (U/L)	29.00(17.00,67.00)	24.00(14.00,43.75) *	28.00(16.00,61.00)	29.00(17.00,72.00) *	31.00(18.00,72.00) *	<0.001
Maximum AST (U/L)	44.00(25.00,108.00)	37.00(23.00,67.75) *	43.00(25.00,102.00)	45.00(26.00,114.25) *	45.00(26.00,113.00) *	<0.001
**Comorbidities**						
Diabetes	4453(31.0 %)	77(15.7 %) *	974(23.2 %)	1361(29.4 %) *	2041(40.4 %) *	<0.001
Renal failure	3585(24.9 %)	99(20.2 %)	983(23.4 %)	1168(25.2 %)	1335(26.4 %) *	<0.001
Congestive heart failure	4988(34.7 %)	141(28.8 %)	1366(32.5 %)	1575(34.0 %)	1906(37.8 %) *	<0.001
Chronic pulmonary disease	3791(26.4 %)	170(34.7 %) *	1035(24.6 %)	1089(23.5 %)	1497(29.7 %) *	<0.001
liver diseases	2983(20.7 %)	89(18.2 %)	813(19.3 %)	914(19.7 %)	1167(23.1 %) *	<0.001

Note: BMI: body mass index; ALT: Alanine aminotransferase; AST: Aspartate aminotransferase.

*: p < 0.05, Normal body weight as the reference.

### 3.2 Crude associations of BMI with outcome variables in patients with different total bilirubin levels

Hospital mortality and hospital and ICU LOSs in patients with different total bilirubin levels are presented in Table 2. Among the patients included in the present study, 18.4 % died in the hospital. Moreover, with an increasing BMI, a decreasing mortality rate was observed. The hospital and ICU LOSs were significantly longer in patients with obesity than in the others (p < 0.05). For patients with total bilirubin levels <2 mg/dL, the mortality rate was 16.0 %. Additionally, the mortality rates of overweight patients and those with obesity were significantly lower than those of normal body weight patients (p < 0.05). Moreover, the hospital and ICU LOSs in patients with obesity were significantly higher than those in normal body weight patients (p < 0.05). The mortality rate of patients with total bilirubin levels ≥2 mg/dL was 29.2 % and that of patients with obesity increased significantly (p < 0.05). The ICU LOS of patients with obesity was significantly higher than that of normal body weight patients (p < 0.05).

**Table 2:** Outcomes of critically ill patients according to bilirubin and BMI categories.

Variables	Total	Underweight	Normal body weight	Overweight	Obesity	p value
**All patients**						
Hospital mortality	2639 (18.4 %)	113(23.1 %)	811(19.3 %)	803(17.3 %)	912(18.1 %)	0.004
Hospital LOS (day)	9.50(5.54,16.86)	9.62(5.67,17.52)	9.05(5.24,15.90)	8.98(5.39,16.33)	10.27(5.84,17.97) *	<0.001
ICU LOS (day)	3.76(2.04,7.44)	3.495(1.90,7.27)	3.49(2.01,6.70)	3.65(2.00,7.02)	4.07(2.15,8.57) *	<0.001
**Patients with total bilirubin** < **2 mg/dL**						
Hospital mortality	1888(16.0 %)	98(22.3 %)	641(18.1 %)	561(14.8 %) *	588(14.6 %) *	<0.001
Hospital LOS (day)	8.98(5.32,15.93)	9.43(5.54,17.53)	8.74(5.05,15.15)	8.71(5.25,15.12)	9.87(5.69,17.01) *	<0.001
ICU LOS (day)	3.69(2.02,7.15)	3.42(1.92,7.21)	3.47(1.99,6.64)	3.57(1.99,6.83)	3.99(2.11,8.14) *	<0.001
**Patients with total bilirubin** ≥ **2 mg/dL**						
Hospital mortality	751(29.2 %)	15(30.0 %)	170(25.7 %)	242(28.5 %)	324(32.0 %) *	0.046
Hospital LOS (day)	11.83(6.63,21.61)	9.84(6.35,16.78)	11.18(6.63,20.60)	11.60(6.33,20.97)	12.74(6.75,22.77)	0.093
ICU LOS (day)	4.08(2.18,8.71)	3.96(1.728,9.04)	3.66(2.11,6.97)	3.94(2.07,8.27)	4.63(2.46,9.91) *	<0.001

Note: BMI: body mass index; LOS: length of stay; ICU: intensive care unit. *: p < 0.05, Normal body weight as the reference.

### 3.3 Adjusted associations of BMI and total bilirubin levels with hospital mortality in critically ill patients

As shown in Table 3, Cox proportional hazard regression analyses were performed to investigate the relationship between different BMI and total bilirubin levels with hospital mortality. In the fully adjusted model 3, the risk of death was significantly decreased in patients with overweightness (HR: 0.869 [0.787, 0.959], p = 0.005) and obesity (HR: 0.876 [0.796, 0.965], p = 0.007) and significantly increased in those with total bilirubin levels ≥2 mg/dL (HR: 1.477 [1.337, 1.632], p < 0.001).

**Table 3:** Cox proportional hazard regression analyses for the hospital mortality of critically ill patients.

Variables	Model 1		Model 2		Model 3	
	HR (95 % CI)	p value	HR (95 % CI)	p value	HR (95 % CI)	p value
BMI						
Normal body weight	reference		reference		reference	
Underweight	1.172 (0.962, 1.427)	0.115	1.189 (0.976, 1.448)	0.086	1.193 (0.979, 1.454)	0.080
Overweight	0.874 (0.793, 0.963)	0.007	0.871 (0.790, 0.961)	0.006	0.869 (0.787, 0.959)	0.005
Obesity	0.823 (0.749, 0.905)	<0.001	0.880 (0.800, 0.968)	0.008	0.876 (0.796, 0.965)	0.007
Total bilirubin						
<2 mg/dL	reference		reference		reference	
≥2 mg/dL	1.462 (1.342, 1.592)	<0.001	1.723 (1.579, 1.880)	<0.001	1.477 (1.337, 1.632)	<0.001

Note: BMI: body mass index; HR: hazard ratio; CI: confidence interval.

Model 1: unadjusted; Model 2: adjusted for gender and age; Model 3: adjusted for gender, age, ALT, AST, and comorbidities (including diabetes, renal failure, congestive heart failure, chronic pulmonary disease, and liver diseases).

### 3.4 Adjusted associations of BMI with outcome variables in patients with different total bilirubin levels

**Fig. 2:**
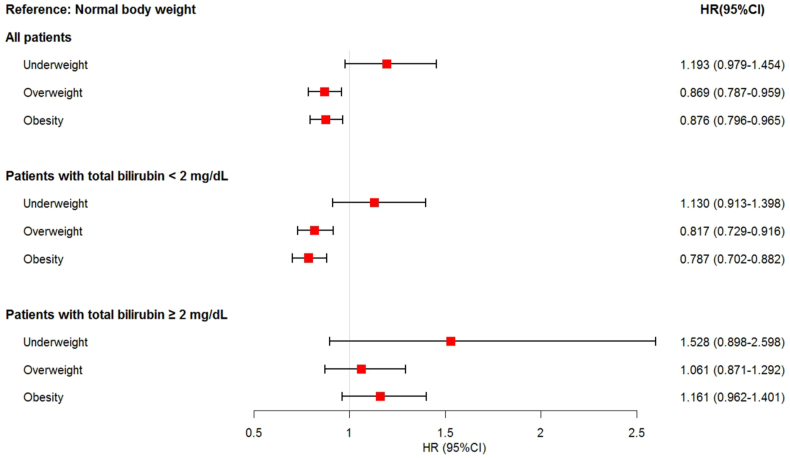
Adjusted hazard ratio for hospital mortality according to bilirubin and BMI categories. All hazard ratios were adjusted for gender, age, ALT, AST, and comorbidities in Cox proportional hazard models. Normal body weight was considered as the reference category.

Cox proportional hazard regression analyses were performed to compare the risk of death in patients with different total bilirubin levels. Patients with normal body weight served as controls. The HRs (95 % CIs) for patients with different bilirubin levels are depicted in Fig. 2. The HRs (95 % CIs) for the underweight, overweight, and obese groups were 1.193 (0.979, 1.454), 0.869 (0.787, 0.959), and 0.876 (0.796, 0.965), respectively. In patients with total bilirubin levels <2 mg/dL, the HRs (95 % CIs) for underweight, overweight, and obese groups were 1.130 (0.913, 1.398), 0.817 (0.729, 0.916), and 0.787 (0.702, 0.882), respectively. In patients with total bilirubin levels ≥2 mg/dL, the HRs (95 % CIs) for underweight, overweight, and obese groups were 1.528 (0.898, 2.598), 1.061 (0.871, 1.292), and 1.161 (0.962, 1.401), respectively.

**Fig. 3:**
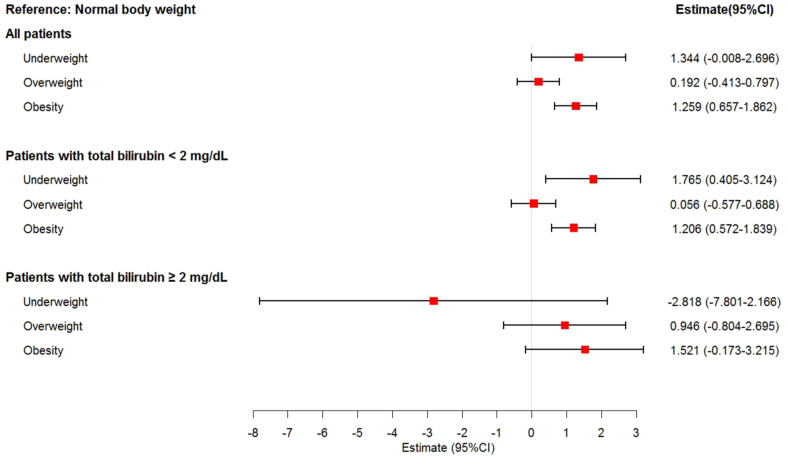
Adjusted estimate for hospital LOS (day) according to bilirubin and BMI categories. All estimates were adjusted for gender, age, ALT, AST, and comorbidities in multivariable linear regression models. Normal body weight was considered as the reference category.

Multivariable linear regression analyses were performed to compare changes in the hospital LOS in patients with different bilirubin levels (Fig. 3). Patients with normal body weight served as controls. For all patients in the underweight, overweight, and obese groups, the estimates (95 % CIs) of the hospital LOS were 1.344 (−0.008, 2.696), 0.192 (−0.413, 0.797), and 1.259 (0.657, 1.862), respectively. In patients with total bilirubin levels <2 mg/dL, the estimates (95 % CIs) of the hospital LOS for the underweight, overweight, and obese groups were 1.765 (0.405, 3.124), 0.056 (−0.577, 0.688), and 1.206 (0.572, 1.839), respectively. In patients with total bilirubin levels ≥2 mg/dL, the estimates (95 % CIs) of the hospital LOS for the underweight, overweight, and obese groups were −2.818 (−7.801, 2.166), 0.946 (−0.804, 2.695), and 1.521 (−0.173, 3.215), respectively.

**Fig. 4:**
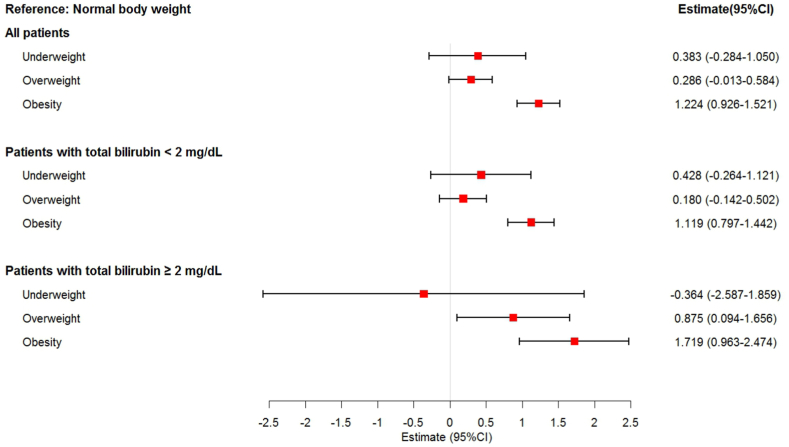
Adjusted estimate for ICU LOS (day) according to bilirubin and BMI categories. All estimates were adjusted for gender, age, ALT, AST, and comorbidities in multivariable linear regression models. Normal body weight was considered as the reference category.

Multivariable linear regression analyses were performed to compare changes in the ICU LOS in patients with different bilirubin levels (Fig. 4). For all patients in the underweight, overweight, and obese groups, the estimates (95 % CIs) of the ICU LOS were 0.383 (−0.284, 1.050), 0.286 (−0.013, 0.584), and 1.224 (0.926, 1.521), respectively. In patients with total bilirubin levels <2 mg/dL, the estimates (95 % CIs) of the ICU LOS for the underweight, overweight, and obese groups were 0.428 (−0.264, 1.121), 0.180 (−0.142, 0.502), and 1.119 (0.797, 1.442), respectively. In patients with total bilirubin levels ≥2 mg/dL, the estimates (95 % CIs) of the ICU LOS for the underweight, overweight, and obese groups were −0.364 (−2.587, 1.859), 0.875 (0.094, 1.656), and 1.719 (0.963, 2.474), respectively.

### 3.5 RCS analyses of BMI with the hospital mortality in patients with different total bilirubin levels

**Fig. 5:**
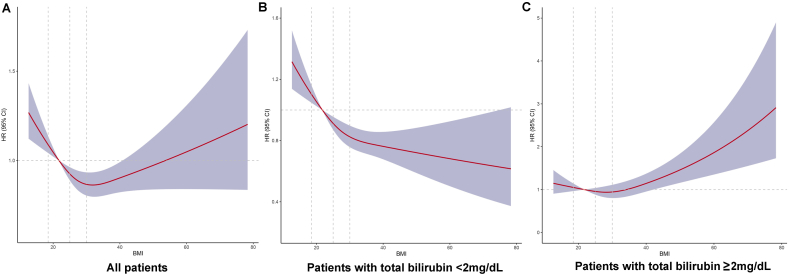
RCS of adjusted hazard ratio between hospital mortality and BMI according to bilirubin categories. A: RCS for all patients; B: RCS for patients with total bilirubin <2 mg/dL; C: RCS for patients with total bilirubin ≥2 mg/dL. RCS models were adjusted for gender, age, ALT, AST, and comorbidities.

RCS analyses were performed to determine the dose–response relationship between BMI and hospital mortality. The risk of death in the present patients gradually decreased with increasing BMI until reaching obesity, after which it increased gradually. The risk of death gradually decreased for patients with total bilirubin levels <2 mg/dL. For patients with total bilirubin levels ≥2 mg/dL, at BMI <30, the risk of death almost did not decrease with increasing BMI; however, as the BMI reached obesity, this risk increased rapidly (Fig. 5).

## 4 Discussion

In this study, we found that the ability of BMI to predict the risk of death greatly differs at different bilirubin levels. Bilirubin levels directly affect the mortality of patients [13] and the predictive ability of BMI. For patients with total bilirubin levels <2 mg/dL, an increased BMI is beneficial to patients’ survival in critical illnesses. For patients with total bilirubin levels ≥2 mg/dL, obesity exerts no protective effect on critically ill patients and increases the risk of death. Regardless of the total bilirubin levels, obesity increases the incidence of hospitalization and ICU LOSs. As total bilirubin levels are an important indicator of liver function, the present results are essential as they show that liver failure can affect the ability of BMI to predict the risk of death in critically ill patients.

Obesity exerted a protective effect on the mortality of critically ill patients in this study, which is consistent with previous reports [4,16]. Generally, patients with obesity have more fat. Additionally, many patients in the ICU are in a state of high carbohydrates and energy consumption [17,18]. The reason behind the protective effect of overweightness and obesity might be that, when compared with normal body weight and underweight patients, those with high-fat content can transform their body fat for supplying carbohydrates and energy to run liver functions normally. The reason for the decreased protective effect of obesity in patients with total bilirubin levels ≥2 mg/dL may be attributed to the importance of the liver in lipid and glucose metabolism [19,20]. However, in the case of liver dysfunction, its fat-transformation ability would be reduced or even lost, and obesity itself would exert certain adverse effects on patients, which may increase the ICU and hospital LOSs.

Although fat facilitates more energy consumption in patients, it may cause more complications in patients with obesity and prolong their hospitalization time. Moreover, the increased incidence of multiple complications is an important adverse effect of obesity [[21], [22], [23]]. Herein, the proportion of patients with obesity accompanied by diabetes, renal failure, congestive heart failure, chronic pulmonary disease, and liver diseases increased significantly, suggesting that obesity increased such complications in critically ill patients. Similarly, although the median total bilirubin levels of patients with obesity did not increase in this study, a change in the bilirubin interquartile range suggested that the proportion of patients with higher bilirubin levels increased with obesity. Obesity increases the risk of fatty liver [24] and cirrhosis [25], thereby resulting in the occurrence of liver diseases and the possibility of liver dysfunction. Nonetheless, for patients with total bilirubin levels <2 mg/dL, the risk of death did not increase in the present study. Conversely, for patients with total bilirubin levels ≥2 mg/dL, obesity increased the risk of death, this could be attributed to the protective effect that outweighs the risk of obesity for patients with normal liver function.

The protective effect of obesity on patients is not invariable. In addition to studies on the effect of bilirubin levels, studies on the effect of age on BMI in predicting death risk are available. The ability of BMI to predict the death risk differs in critically ill patients of different ages [26]. Additionally, a subgroup analysis of BMI regulating the mortality of patients with sepsis revealed that, when compared with patients aged >65 years, those <65 years of age showed a poor protective effect of obesity on mortality [27]. Thus, the protective effect of obesity differs for different ages and bilirubin levels.

In addition to being an indicator of liver injury, bilirubin exerts anti-inflammatory [28] and antioxidant effects [29]. For patients with obesity, adipose tissues are associated with inflammation [30,31] and oxidative stress [32]. However, the existing research is inadequate to confirm whether bilirubin plays a regulatory role. The main mechanism underlying the obesity-based prediction of the survival of critically ill patients with different bilirubin levels may be attributed to the role of bilirubin as a marker for liver function.

Nevertheless, the study has several limitations. First, we used data extracted only from the MIMIC IV database, which limits the tested dataset. For instance, the database does not provide information about the specific circumstances of death for the patients. Second, as an index to evaluate the nutritional status of patients, BMI has some shortcomings. For instance, increased BMI due to increased muscle tissues cannot reflect the degree of obesity of patients. Similarly, increased BMI due to edema mass cannot reflect the nutritional status of patients. Third, among the patients we included, the number of underweight patients was less. Thus, owing to the limitation of the number of patients, those with obesity were not graded. Fourth, this was a retrospective analysis, and the mechanism underlying the effect of bilirubin levels on the predictive ability of BMI was not elucidated. Despite these limitations, we investigated differences in the ability of BMI to predict the risk of death in critically ill patients with different bilirubin levels.

## 5 Conclusions

The ability of BMI to predict the risk of death in patients with different bilirubin levels greatly differs, which suggests that liver fat metabolism plays a crucial role in the survival of critically ill patients. Future studies should investigate the probable role of liver fat metabolism in critically ill patients.

## Funding

This research did not receive any specific grant from funding agencies in the public, commercial, or not-for-profit sectors.

## Ethics approval and consent to participate

Review and/or approval by an ethics committee was not needed for this study because data used were extracted from an open source databases and were anonymized.

## Data availability statement

Data analyzed in the present study are available in the MIMIC database Ⅳ (https://www.physionet.org/content/mimiciv/2.0/).

## CRediT authorship contribution statement

**Xiao-Ling Lv:** Writing – review & editing, Writing – original draft, Visualization, Methodology, Formal analysis, Data curation, Conceptualization. **Ying-Xing Yue:** Writing – review & editing, Visualization, Data curation. **Bing-Bing Jia:** Writing – review & editing, Methodology, Conceptualization. **Ying-Zheng Weng:** Writing – review & editing, Visualization, Methodology. **Yan Lu:** Writing – review & editing, Visualization, Data curation. **Zhou-Xin Yang:** Writing – review & editing, Writing – original draft, Methodology, Formal analysis, Data curation, Conceptualization.

## Declaration of competing interest

The authors declare that they have no known competing financial interests or personal relationships that could have appeared to influence the work reported in this paper.
